# A Conjugation Strategy to Modulate Antigen Binding and FcRn Interaction Leads to Improved Tumor Targeting and Radioimmunotherapy Efficacy with an Antibody Targeting Prostate-Specific Antigen

**DOI:** 10.3390/cancers13143469

**Published:** 2021-07-11

**Authors:** Oskar Vilhelmsson Timmermand, Anders Örbom, Mohamed Altai, Wahed Zedan, Bo Holmqvist, Marcella Safi, Thuy A. Tran, Sven-Erik Strand, Joanna Strand

**Affiliations:** 1 Department of Clinical Sciences Lund, Oncology, Lund University, 22243 Lund, Sweden; oskar.vilhelmsson_timmermand@med.lu.se (O.V.T.); anders.orbom@med.lu.se (A.Ö.); mohamed.altai@igp.uu.se (M.A.); wahed.zedan@med.lu.se (W.Z.); marcella_elin.safi.2028@student.lu.se (M.S.); sven-erik.strand@med.lu.se (S.-E.S.); 2 ImaGene-iT AB, Medicon Village, 22363 Lund, Sweden; bo@imagene-it.se; 3 Department of Radiopharmacy, Karolinska University Hospital, 17177 Stockholm, Sweden; thuy.tran@ki.se; 4 Department of Clinical Sciences Lund, Medical Radiation Physics, Lund University, 22243 Lund, Sweden; 5 Department of Clinical Oncology, Skane University Hospital, 22243 Lund, Sweden

**Keywords:** prostate cancer, FcRn, radionuclide therapy, hu5A10, PSA

## Abstract

**Simple Summary:**

The present study demonstrates that a radiolabeling strategy can positively modify hu5A10′s capacity to bind PSA and complex with the FcRn receptor, which results in a more homogenous activity distribution in tumors and enhanced therapy efficacy. These new innovative radiochemistry ideas could potentially decrease relapse of tumors in patients treated with internalizing antibodies.

**Abstract:**

Background: The humanized monoclonal antibody (mAb) hu5A10 specifically targets and internalizes prostate cancer cells by binding to prostate specific antigen (PSA). Preclinical evaluations have shown that hu5A10 is an excellent vehicle for prostate cancer (PCa) radiotheranostics. We studied the impact of different chelates and conjugation ratios on hu5A10′s target affinity, neonatal fc-receptor interaction on in vivo targeting efficacy, and possible enhanced therapeutic efficacy. Methods: In our experiment, humanized 5A10 (hu5A10) was conjugated with DOTA or DTPA at a molar ratio of 3:1, 6:1, and 12:1. Surface plasmon resonance (SPR) was used to study antigen and FcRn binding to the antibody conjugates. [^111^In]hu5A10 radio-immunoconjugates were administered intravenously into BALB/c mice carrying subcutaneous LNCaP xenografts. Serial Single-photon emission computed tomography (SPECT) images were obtained during the first week. Tumors were harvested and radionuclide distribution was analyzed by autoradiography along with microanatomy and immunohistochemistry. Results: As seen by SPR, the binding to PSA was clearly affected by the chelate-to-antibody ratio. Similarly, FcRn (neonatal fc-receptor) interacted less with antibodies conjugated at high ratios of chelator, which was more pronounced for DOTA conjugates. The autoradiography data indicated a higher distribution of radioactivity to the rim of the tumor for lower ratios and a more homogenous distribution at higher ratios. Mice injected with ratio 3:1 ^111^In-DOTA-hu5A10 showed no significant difference in tumor volume when compared to mice given vehicle over a time period of 3 weeks. Mice given a similar injection of ratio 6:1 ^111^In-DOTA-hu5A10 or 6:1 ^111^In-DTPA-hu5A10 or 12:1 ^111^In-DTPA-hu5A10 showed significant tumor growth retardation. **Conclusions:** The present study demonstrated that the radiolabeling strategy could positively modify the hu5A10′s capacity to bind PSA and complex with the FcRn-receptor, which resulted in more homogenous activity distribution in tumors and enhanced therapy efficacy.

## 1. Introduction

Prostate cancer (PCa) is the most common cancer in men in the Western world [1]. One of every 8–10 patients diagnosed with PCa will die from this disease. There is currently no curative treatment for patients with metastatic castration-resistant prostate cancer (mCRPC), despite an expanding arsenal of targeted agents to treat and monitor the disease. Existing therapies have limited treatment efficacy with a minor increase in prolonged life expectancy [2]. As prostate cancer is dependent on androgen receptor (AR) pathway signaling for proliferation, relapse following androgen deprivation therapy relies on reactivation of the AR or crosstalk between the AR and other signal transduction pathways. Treatments of prostate cancer are currently focusing on targets downstream of the androgen receptor (AR) pathway such as prostate-specific membrane antigen (PSMA) [3]. One promising strategy to manage metastatic PCa is targeted radionuclide therapy [4]. Both lutetium-177-labeled PSMA-targeted antibodies (J591) and small molecules have shown promising anti-tumor effects, albeit often transitory and with decreasing therapeutic efficacy upon re-dosing. It is known that AR signaling results in down-regulation of FOLH1, the gene coding for PSMA (folate hydrolase 1, FOLH1), and subsequently a lower expression of PSMA. This has presented a challenge, as it impacts the diagnostic readout for PSMA ligand positron emission tomography (PET).

Prostate-specific antigen (PSA, kallikrein-related peptidase 3, *KLK3*) is another interesting target for prostate cancer radiotheranostics downstream of the AR. PSA is uniquely and abundantly expressed in healthy and malignantly derived prostate tissues, which decreases risks for false-positive diagnostic findings and side-effects related to uptake in non-prostatic tissues [5,6,7]. Several pathological processes, such as malignancy and inflammation, cause retrograde release of free PSA (fPSA) into the blood circulation. When this occurs, serine protease inhibitors (such as alpha(1)-antichymotrypsin, ACT) immediately form a stabile complex with PSA by binding to the catalytic cleft. The PSA concentration in blood is approximately a millionth-fold lower than in prostate tissues.

We have previously reported on development of non-invasive fPSA-targeted radiotheranostics, including PET-based detection of metastatic disease, assessment of lesion specific AR-activity, and prostate-tumor-specific delivery of both alpha and beta radionuclides [8,9,10]. Our technology is based on an IgG1 antibody (5A10) that selectively targets tissue-associated fPSA; 5A10 binds to an epitope specific to the catalytic cleft of fPSA, which effectively avoids binding to forms in the blood circulation. After binding to fPSA, 5A10 is permanently internalized into the target cell by a neonatal fc-receptor-driven process (FcRn) [11]. FcRn is a MHC-I related protein that takes part in the transport of IgGs; i.e., transcytosis across cells into the circulation via the neonate gut of rodents, or similarly in humans via the placenta, to the circulation of the prenate. FcRn is also involved in IgG recycling and its expression in the endothelial cells of the vasculature is believed to regulate IgG perseverance. Another important location of FcRn expression is the liver, also believed to be an FcRn-driven site for IgG recycling, with expression in early, late, or recycling endosomes, rather than in the trans Golgi network or lysosomes. The result of recycling is a prolonged IgG half-life, an important immunological feature contingent upon the IgG-type’s fc-portion and its ability to bind to the neonatal fc-receptor. If IgGs are unable to do so they are mainly catabolized in the liver.

An absolute majority of the radiolabeling methods applied for antibodies rely on conjugating the chelates to random lysine residues on the antibody. Chelates may become appended to regions on the fc or the Fab that is crucial to the immunological mechanisms described above. Edelmann et al. have reported that high conjugation ratios of chelator to mAb may reduce the affinity of IgGs to FcRn. This is believed to influence the lysosomal degradation of the antibody, particularly in endothelial cells [12].

It is known from studies of therapeutic antibodies, radioimmunoconjugates, and antibody drug conjugates that antibodies with high affinity towards their antigen, like hu5A10, do not necessarily have high therapeutic efficacy, as a high affinity negatively impacts the penetration into a tumor mass. We have earlier shown that biokinetics is heavily dependent on conjugation strategy for small molecules [13,14,15]. In the current study, we have evaluated the influence of an increasing number of the p-SCN-CHX-A″-DTPA or p-SCN-Bn-DOTA chelators per hu5A10 mAb with regards to fPSA binding, FcRn interaction, tumor penetration, and blood retention. The Auger electron emitter ^111^In (mostly emitting Auger electrons with only conversion electrons at 144.5 keV in 8% of decays, and at 218.6 keV in 5% of decay, and with its therapeutic effect highly dependent on cellular internalization) was used. We evaluated the efficacy of radionuclide therapy using ^111^In-hu5A10 to determine if a more homogenous tumor activity and thus absorbed dose distribution could be achieved through careful modification of the conjugation chemistry. Furthermore, a study with ^177^Lu-labeled DTPA-hu5A10 ratio 12:1 was performed in mice bearing 22RV1 xenografts both to compare the uptake with LNCaP xenografts and to investigate the feasibility of ^177^Lu labeling for future therapy studies.

## 2. Results

### 2.1. SPR

The affinity (KD) of DTPA-conjugated hu5A10 was lower than that of DOTA-conjugated hu5A10 (Table 1). Further, a decrease in FcRn binding, measured as the ratio of antibody to FcRn on the chip, with increasing DOTA-chelation indicated that an increased number of chelates on the fc-part could reduce fc-FcRn interaction. Kallikrein-related peptidase 2, immobilized in an adjacent flow cell, was used as control, and no binding of the PSA specific antibody or its immunoconjugates was detected.

### 2.2. Conjugation of hu5A10 and Radiolabeling

The number of DTPA and DOTA molecules per antibody was determined by labelling of the (DTPA)X-hu5A10 and (DOTA)X-hu5A10 conjugates with different ratios of ^111^InCl_3_/^nat^InCl_3_ as described above. Results showing the average chelator-to-hu5A10 ratio for each conjugate are presented in Table 2.

Results from the radiolabeling experiments revealed that it was possible to label DTPA immunoconjugates with ^111^In and radiochemical yields exceeding 85%. However, DOTA conjugates demonstrated significantly lower radiochemical yields, 74% ± 3.0 for ratio 3:1, and 77% ± 6.0 for ratio 12:1, potentially reflecting the slow kinetic for DOTA and/or the comparatively fewer chelators per antibody (Table 2). Radiolabeling of the immunoconjugate CHX-A″-DTPA-hu5A10 with ^177^Lu was successful, with a radiochemical yield exceeding 90%.

### 2.3. In Vivo SPECT/CT Imaging

**Figure 1:**
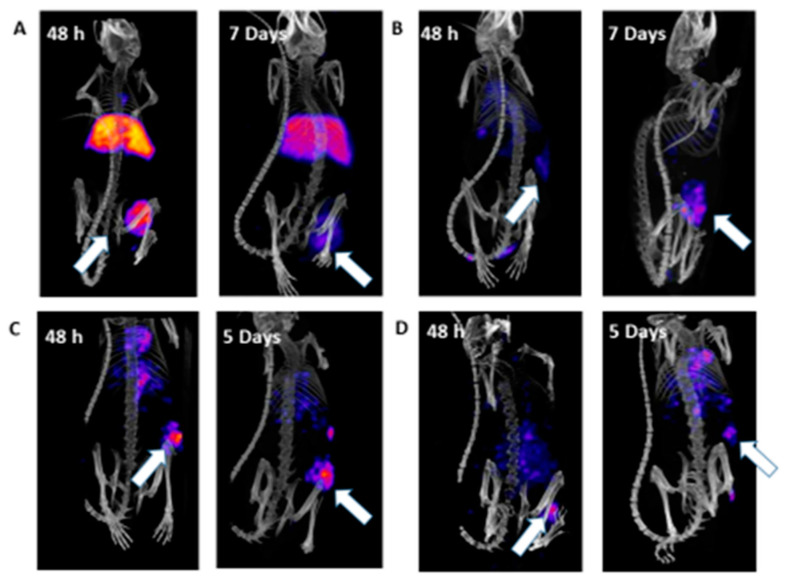
Effect on activity distribution of chelator and chelate-to-antibody ratio. (**A**) ^111^In-DTPA-hu5A10 ratio 3:1 at 48 h and 7 days; (**B**) ^111^In-DTPA-hu5A10 ratio 12:1 at 48 h and 7 days; (**C**) ^111^In-DOTA-hu5A10 3:1 at 48 h and 5 days; (**D**) ^111^In-DOTA-hu5A10 ratio 12:1 at 48 h and 5 days. There was a large shift in activity distribution for DTPA, but not as large as a difference as was seen for ^111^In-DOTA-hu5A10. This can be seen in a clear shift from high liver uptake for ratio 3:1 to high tumor uptake for ratio 12:1.

For DTPA-labeled conjugates, there seemed to be a shift from high liver uptake at low ratios to low liver uptake at a high ratio (Figure 1A,B). For ^111^In-DOTA-hu5A10 ratio 3:1 and ratio 6:1, the shift was not as pronounced (Figure 1C,D), which could have been due to a lower affinity to FcRn for DOTA-hu5A10 compared to DTPA-hu5A10. Instead, there was a relatively higher tumor uptake (e.g., a tumor-to-liver ratio of 0.61 ± 0.18 for ratio 3:1 versus 0.82 ± 0.08 for ratio 12:1 at 24 h). Interestingly, at later timepoints (120 h and 168 h), the 12:1 conjugation ratio had a higher tumor-to-liver ratio than ratio 3:1 for both DOTA- and DTPA-conjugated hu5A10 (0.78 ± 0.06 versus 1.05 ± 0.19 for DOTA and 0.3 ± 0.1 versus 1.7 ± 0.3 for DTPA). All tumor-to-blood and tumor-to-liver ratios can be found in Table 3.

For ^111^In-DOTA-hu5A10, tumor-to-blood showed an increased ratio with time, and the conjugation ratios of 6:1 and 12:1 showed the highest values at both 48 h and 5 days post-injection. The tumor-to liver-ratio was significantly higher for ratio 6:1 compared to ratio 3:1 at both 48 h and 5 days post-injection. Ratio 6:1 demonstrated the most favorable tumor-to-organ ratios. At 5 days post-injection, ratio 6:1 had significantly higher tumor-to-liver ratios compared to ratio 3:1. The chelator-to-antibody ratio 12:1 had significant higher tumor-to-liver ratio compared to 3:1 at 5 days.

For ^111^In-DTPA-hu5A10, the tumor-to-blood ratio was higher for the 3:1 conjugation ratio, whereas the tumor-to-liver showed the opposite, with a significantly higher tumor-to-liver ratio at 7 days post-injection for ratio 12:1 compared to 3:1.

**Figure A1:**
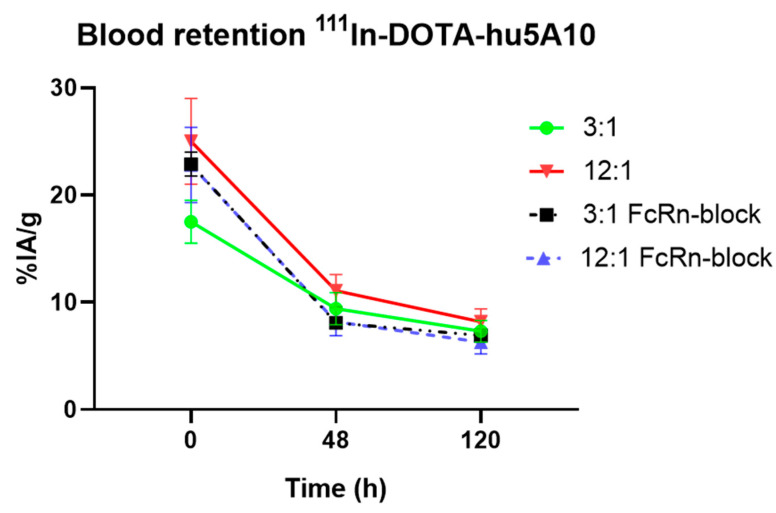
Blood retention for ^111^In-DOTA-hu5A10 with 3:1 and 12:1 chelate-to-antibody molar ratios. The results for FcRn blocking are also included. As can be seen, there was an accelerated blood clearance observed for ratio 3:1. FcRn blocking enhanced the blood retention for both ratios. Results from SPECT imaging performed at 0, 2, and 5 days post-injection.

There was an increased blood concentration for ratio 12:1 compared to ratio 3:1, FcRn blocking enhanced the blood retention for both ratios, and there was a significant higher blood retention for ratio 3:1 after FcRn blocking (Figure A1).

### 2.4. Uptake Study in 22RV1 Xenografts

The tumor-to-liver ratio for 22RV1 xenografts was consistent with LNCaP (1.6 ± 0.2 vs. 1.7 ± 0.3) at 7 days p.i. The liver uptake for 22RV1 was in agreement with the liver uptake of LNCaP (4.0 ± 0.8%IA/g vs. 5.6 ± 0.9%IA/g). The tumor uptake was in well agreement with previously obtained results using ^89^Zr-labeled 5A10 in 22Rv1 xenografts [8].

### 2.5. Autoradiography, Immunohistochemistry, and Immunofluorescence

**Figure 2:**
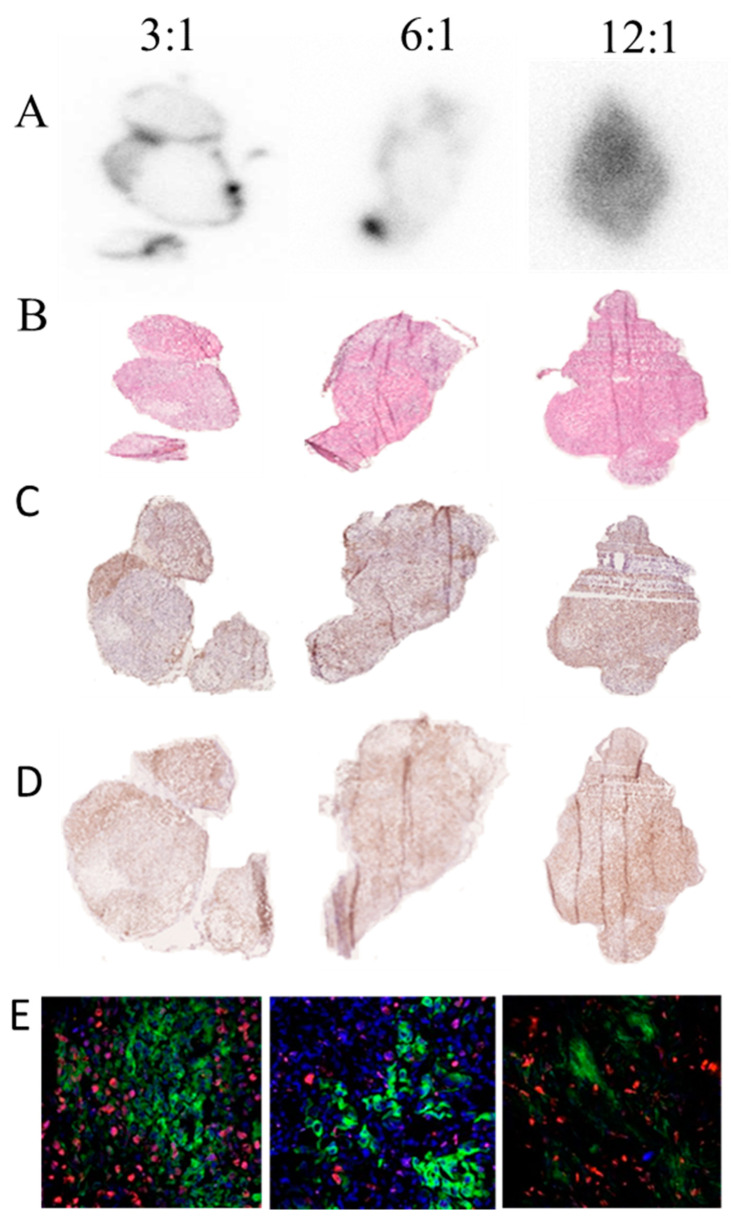
Digital autoradiography and immunohistochemistry of ^111^In-DOTA-hu5A10 5 days after injection. (**A**) Each representative digital autoradiography image of activity uptake is individually scaled from 0 (white) to max (black) signal in that section, demonstrating that the higher the chelate-to-antibody molar ratio, the more even the activity distribution in the tumor. (**B**) The same tissue sections as in (**A**) stained with hematoxylin and eosin, demonstrating the tumor histology. (**C**) Adjacent sections illustrating the distribution of immunohistochemically labelled Ki-67-positive proliferating cells. (**D**) Adjacent sections to (**C**), demonstrating the distribution of immunohistochemically labelled PSA-positive cells. (**E**) Immunofluorescence double labeling, illustrating the distributional relation of radionuclide uptake primarily in viable tumor regions with PSA-expressing cells (green) and Ki67-positive cells (red), rather than in necrotic regions.

In the 3:1 chelate-antibody ratio group (Figure 2), there was a high radionuclide uptake in junctions between nodules in the tumor sections. These areas contained binding tissue, but also a quite dense concentration of viable tumor cells. Additionally, there was moderate uptake around most of the edges of the tumor nodules that had a denser concentration of viable tumor cells than the inside of the nodules. The tumor distribution of higher levels of activity uptake correlated well with a high Ki67 immunohistochemical labeling. Incongruently, there was scarce Ki67 labeling and almost absent radionuclide uptake at the center of the tumor nodules. There was also some variation in the degree of correlation between the radionuclide and Ki67 distribution between individual nodules in the same tumor sections.

PSA-immunolabelled adjacent sections showed high levels of PSA labeling between and along the edges of tumor nodules, which correlated with the distribution of the radionuclide uptake hotspots. However, central areas of the tumor nodules had low levels of both PSA labeling and radionuclide uptake.

In the 6:1 chelator-to-antibody ratio group, the tumor distribution of radionuclide uptake coincided with the higher density of viable cells, but also showed variations with high radionuclide uptake along the edges comprising a mix of viable tumor cells and connective tissue. The intratumoral distribution of high radionuclide uptake and Ki67 labeling correlated to some degree, but also differed between sections; i.e., between levels of the tumors. In central areas, the Ki67 labeling correlated with a lower degree of radionuclide uptake. There was a corresponding distributional correlation of radionuclide uptake and PSA labeling, such as in areas along the edges of the tumor nodules, and PSA labeling correlating with lower radionuclide uptake in central parts of each nodule.

In the 12:1 chelate-to-antibody ratio group, the radionuclide uptake was more evenly distributed throughout the whole tumor nodule, and there was no correlation between a higher density of tumor cells and higher uptake. Some sections even showed an inverse correlation. In addition, there was no correlation between the general pattern of radionuclide uptake and Ki67-labeled regions. There was, however, a high degree of distributional correlation of PSA labeling and radionuclide uptake in most parts of the tumor nodules.

These results showed that a higher chelate-to-antibody ratio provided a more homogenous radioactivity distribution, which resulted in a more homogenous absorbed dose distribution, and thereby a larger volume of the tumor receiving a similar therapeutic absorbed dose.

The immunofluorescence labeling (Figure 2) confirmed the distributional correlation of hu5A10-targeted, PSA-labeled cells with regions with ongoing cell proliferation (Ki67-labeled), as indicated by the single labeling in adjacent sections (above). The double labeling further supported the distributional relation of radionuclide uptake in regions with viable rather than necrotic cells; i.e., comprising PSA-expressing cells and proliferating (Ki67-positive) cells. The PSA-labeled cells were not Ki67-positive, and thus comprised separate cellular populations, located in close vicinity to each other, intermingled in some regions, or more separated in other regions of the tumors.

The antibody control sections showed no labeling, which supported the specific binding by both the primary and secondary antibodies to their individual epitopes.

### 2.6. Therapeutic Efficacy

**Figure 3:**
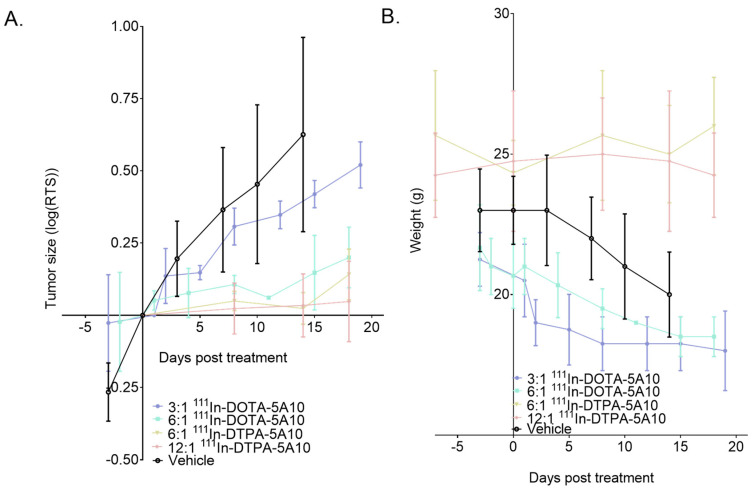
The therapy efficacy for ^111^In-DOTA-hu5A10 and ^111^In-DTPA-hu5A10 for chelate-to-antibody ratios of 3:1, 6:1, and 12:1. (**A**) Tumor size ratio (log(RTS)) and (**B**) weight change during therapy. It can be clearly seen that the therapy efficacy was highly dependent on the chelate-to-antibody molar ratio and chelator.

Results for the therapeutic efficacy of ^111^In-DOTA-hu5A10 and ^111^In-DTPA-hu5A10 with chelate-to-antibody ratios of 3:1, 6:1, and 12:1 are displayed in Figure 3. Data are presented as changes in relative tumor size ratio (log(RTS)), as well as weight changes during therapy, for the different therapy groups It can be clearly seen that the therapy efficacy was highly dependent of the choice of chelator and chelate-to-antibody molar ratio.

Mice injected with circa 18 MBq and chelate-to-antibody ratio 3:1 ^111^In-DOTA-hu5A10 showed no significant difference in tumor volume when compared to mice given vehicle over a time period of 3 weeks. However, mice given a similar injection of chelate-to-antibody ratio 6:1 ^111^In-DOTA-hu5A10 or 6:1 ^111^In-DTPA-hu5A10 or 12:1 ^111^In-DTPA-hu5A10 showed significant tumor growth retardation, as seen in Figure 3A when plotting the relative tumor size with regards to the day of injection. Further, mice given ^111^In-DTPA-hu5A10 showed little to no change in body weight over this time course, indicating that the treatment was well tolerable (Figure 3B). As the health of mice given only vehicle deteriorated with increasing tumor burden, as well as for mice given ^111^In-DOTA-hu5A10, they subsequently lost weight, and consequently the therapy study was ended about three weeks post-injection.

## 3. Discussion

Monoclonal antibody (mAb)-based therapeutics are an established and clinically successful way of treating cancer [16]. The success or demise of a therapeutic mAb is not just reliant on its specificity towards the target. Unlike small-protein-based targeting agents, mAbs are bulky agents and hence are less prone to modifications induced by payload coupling. However, multiple reports have been published on the influence of conjugation ratio on mAbs properties [17,18,19,20,21]. Higher chelator-to-antibody conjugation ratios may alter physical and biological properties of the mAb. One example is the decrease in binding affinity of the conjugated mAb to the target of interest. Pharmacokinetics may also be compromised due to modification of the overall charge (isoelectric point) of the mAb, and increased recognition of the excessively decorated mAb by the reticuloendothelial system present in the liver and spleen. Further, Edelmann et al. [12] have recently shown, using affinity chromatography, that conjugation of IgGs with bifunctional chelators, intended for labeling with radiometals, as well as direct radioiodination, interfered with the FcRn–antibody binding.

Herein, we have shown that conjugation methodology of hu5A10 had an impact on the in vitro properties; i.e., both FcRn and PSA binding, of the immunoconjugates, as well as the in vivo distribution of hu5A10, tumor distribution, and therapy efficacy. The tumor and tissue uptake and ratios for the two xenograft types LNCaP and 22RV1, with 22RV1 being a cell line with lower basal expression of fPSA, were similar and in well agreement with previous results with ^89^Zr-labled 5A10 in 22Rv1 and LNCaP-AR xenografts [8]. However, we observed differences in blood and organ concentrations of radioactivity depending on the conjugation ratio. Interestingly, the intratumoral distribution of the radioimmunoconjugate, as well as therapy outcome, were highly influenced by the chelator-to-antibody ratio.

The tendency of mAbs and their conjugates to distribute in a non-homogenous fashion inside the tumor volume, a result of strong interaction with antigen on cells in the outer layers of tumors, leading to low tumor penetration, is denoted by the binding-site barrier (BSB) [22]. The BSB is believed to contribute to heterogeneous activity and absorbed dose distributions with a positive therapy effect in regions with high uptake, but with the risk of relapse in parts of the tumor that receive sub-therapeutic levels of the antibody or antibody conjugate [22,23,24,25,26]. ^111^In-DOTA-hu5A10 conjugated antibodies produced with the higher conjugation ratio were less prone to bind FcRn and PSA in vitro, and the corresponding radioimmunoconjugate had a significantly more homogenous intratumoral distribution as compared to the lower ratios (Figure 2). This could be due to overcoming of the BSB following reduced binding to PSA, but also potentially because of weaker binding to, and thus less frequent internalization via, FcRn. All together, this allows increased penetration into the tumor mass.

However, there are other parameters that affect tumor uptake, specifically, blood concentration. For DOTA-conjugated hu5A10, which had a significant reduction in the FcRn binding when increasing the conjugation ratio, there was a higher blood retention for ratio 12:1 than for ratio 3:1 at 0 h (Figure A1). As diffusion is a main driver of tumor accumulation [27,28,29,30], a higher systemic availability of these radioimmunoconjugates, not being shuttled by the FcRn, could be the reason that they ended up with a more beneficial tumor-to-liver profile than those conjugated at 3:1. This could have implications for both therapy outcome and absorbed doses to normal organs.

However, blocking of the FcRn receptors in vivo is stoichiometrically unfeasible, but saturating FcRn did significantly affect the blood retention for ratio 3:1, with increased blood levels at 0 h, which was not seen for ratio 12:1 (Figure A1). Both DOTA- and DTPA-conjugated hu5A10, despite the small effect in vitro on FcRn binding with DTPA (Table 1), had a better tumor-to-liver ratio at higher ratios (Table 3). This indicated that antibodies with retained FcRn binding were trapped in the liver, an organ with high FcRn expression, to a larger degree than radioimmunoconjugates with reduced FcRn binding.

For DTPA conjugation, the tumor-to-liver ratio had a fivefold increase for ratio 12:1 compared to ratio 3:1 (Table 3), and similar tendencies were observed earlier for antibodies with a high number of conjugated chelates [13] (see Table 2). The change in antibody–antigen kinetics inside the tumor, with a lower affinity to PSA (a KD 10 times larger than that for DOTA-conjugated antibodies) could lessen the effects of the BSB. In vitro, binding to the FcRn was mostly retained when conjugating with DTPA (Table 2). This combined, efficient penetration and clearance via tumor cell internalization could drive tumor accumulation and lead to the larger differences seen. Mass spectrometry of the DOTA- and DTPA-labeled antibodies revealed that the difference seen with respect to FcRn binding between the two chelates could potentially be explained by differences in placement of the chelates, even though the reaction chemistry was the same (unpublished data).

To test the hypothesis that the differences seen herein affected the outcome when applied to radioimmunotherapy, DOTA-hu5A10 and DTPA-hu5A10 were labeled with indium-111 (^111^In). ^111^In is a radionuclide mostly used for imaging, as it emits two gammas, 171 keV (90%) and 245 keV (94%), and K-X-rays 23–26 keV (82%) when it decays to cadmium-111. It can also be used for therapy because of its large emission of Auger electrons (K (119 keV/16%), L (2.7 keV/98%)) and conversion electrons (124–245 keV/15%). Therapeutic effects have been seen when coupled to internalizing and residualizing targeting agents [31]. The short range (nm) of the electrons means that they have no crossfire effect. Hypothetically, ^111^In should thus be an excellent radionuclide if one wants to reveal the differences in outcome one could expect from heterogeneous and variable uptake. Our results showed that there seemed to be such an effect (Figure 3). The change in uptake as ratio of tumor-to-blood or directly in activity concentration seemed to be reflected in the therapeutic outcome. Surprisingly, there were no significant differences between the two different chelates. The bystander effect could be a (probably minor) explanation for the observed therapy effect, in which a more homogenous activity distribution, probably not targeting all cells but cells in close vicinity, could lead to a bystander effect.

There are several other factors that could affect the distribution in the tumors, such as complexation or charge of the immunoconjugate. When comparing the biokinetics for DTPA-hu5A10 ratio 12:1 with previously published biokinetic results for DTPA-hu5A10 ratio 3:1, we could not find any differences in macroscopic tumor uptake—thus the tumor mean absorbed dose might not be the reason for the better therapy effect. The main objective of this study was to show that conjugation ratios altered the intratumoral distribution. We further used ^111^In as proof of concept for any effect on the therapy efficacy and as an indicator of internalization of the antibody. As explained above, the Auger electrons emitted from indium-111 can only have a therapeutic effect if it is in close proximity to the DNA. We have previously shown that antibodies that bind to secreted antigens as PSA are internalized into the cell due to complexation with FcRn [11]. Here, we used two conjugation strategies, one leading to reduced FcRn binding and one to reduction in binding of PSA, as shown in vitro. Both DOTA- and DTPA-conjugated hu5A10 performed better when conjugated at higher ratios. This could be due to overcoming the binding site barrier (lower affinity to PSA or FcRn). Collectively, the results herein will provide a radioconjugate with favorable characteristics for further radioimmunotherapy studies.

## 4. Materials and Methods

### 4.1. SPR

The binding interaction between the hu5A10 immunoconjugates, recombinant PSA (Innovagen AB, Lund, Sweden, DiaProst, Lund, Sweden), and FcRn was studied by surface plasmon resonance (SPR) (Biacore 2000 system, GE Healthcare, Boston, USA). Human kallikrein-related peptidase 2 (hK2, *KLK2*) was used as control for PSA. Antigens were immobilized on a CM4 research grade chip (GE Healthcare) using a covalent amine coupling kit (GE Healthcare). Different concentrations (100, 50, 25, 12.5, and 10 nM) of the conjugated and unconjugated variants of antibody were flown over the immobilized channels with a flow rate of 30 μL/min. The association phase of the immunoconjugate was followed for 4–5 min, and the dissociation phase was extended up to 480 min. To determine the binding of FcRn to the hu5A10-PSA complex, 10 min into the dissociation phase, 40 nM FcRn (Acrobiosystems Inc, Newark, DE, USA) in 10 mM maleic acid (pH 6.0) buffer was flown over the chip, containing the coupled antigen and bound antibody, for 3 min. Thereafter the dissociation phase was resumed. The output signals generated by the blanks were subtracted from the other flow cells. The affinity of each immunoconjugate was calculated by fitting a bi-exponential function to the binding and the dissociation curves. FcRn binding was evaluated by calculating the number of FcRn bound per antibody on the chip at the end of FcRn injection. Response units (RUs) corresponding to bound antibody on the chip just before the end of FcRn injection were calculated by intrapolation using the dissociation phase before and after the injection of FcRn. This was possible because of the highly pH dependent nature of FcRn binding. Knowing the RUs for both the antibody and FcRn at the end of the FcRn injection, as well as the molar mass of both molecules, the ratio of FcRn to antibody could then be calculated based on the assumption that RUs directly correlated to the mass on the chip.

### 4.2. Conjugation of hu5A10 and Radiolabeling

Humanized 5A10 (hu5A10) (Innovagen AB, Lund and Diaprost) was conjugated with either CHX-A″-DTPA (Macrocyclics, Plano, TX, USA) or p-SCN-Bn-DOTA (DOTA) (Macrocyclics) at a 3:1, 6:1, or 12:1 chelator-to-antibody molar ratio. The conjugates were stored at −8 °C and used/labeled within the same day of conjugation (see Appendix A).

Labeling efficiency of the radiolabeled conjugates was evaluated using a previously established thin-layer chromatography protocol, which utilized a phosphor imager system and Optiquant software (all details are given in the Appendix A).

The average number of chelators conjugated to each mAb was determined according to Mears et al. [32]. In brief, the immunoconjugate was mixed with a ^111^InCl_3_/^nat^InCl_3_ solution consisting of known concentrations of non-radioactive (Sigma-Aldrich, Saint-Louis, MO, USA) and radioactive (^111^In) indium. The radioactive yield was, together with the assumption of a 1:1 interaction between indium atoms and chelators, utilized for calculating the number chelates attached to each mAb.

### 4.3. In Vivo SPECT/CT Imaging

All animal experiments were performed in accordance with national legislation on laboratory animal protection and permitted by the Local Ethics Committee for Animal Research at Lund University (ethical permission number 4350-20).

A 200 µL 1:1 cell suspension of Matrigel (Corning) and 5–7 × 106 LNCaP cells (Prostate Carcinoma cells Clone FGC ATCC^®®^CRL-1740, Lot 5972254), Rv22 in RPMI 1640, were unilaterally implanted subcutaneously (s.c.) on the right hind leg of male BALB/cnu/nu mice. Animals were regularly monitored for tumor growth, body weight, and physical signs of illness. If the tumor diameter reached >15 mm, or if a severe decline in general condition was noticed, animals were immediately euthanized.

The SPECT imaging was performed using a preclinical SPECT/CT (see Appendix A).

To specifically evaluate if saturating the FcRn receptor would affect the blood retention of ^111^In-p-SCN-Bn-DOTA-hu5A10 at ratios 3:1 and 12:1, 800 µg of Fc fragment (ab90285, Native Human IgG FC fragment protein, GR3276085, Abcam, Cambridge, United Kingdom) was administered by intraperitoneal injection directly before the radiotracer was injected (30μg per mouse, i.v, 10–13 MBq) in LNCaP-bearing BALB/cnu/nu mice (*n* = 3–4). Each animal was anesthetized at 0 and 48 h and 5 days post-injection, and SPECT imaging was conducted. Activity uptake in the heart was quantified as a percentage of the injected activity minus activity remaining in the tail.

To specifically evaluate the effect of FcRn blocking on blood retention of ^111^In- p-SCN-Bn-DOTA-hu5A10 at ratios 3:1 and 12:1, 800 µg of Fc fragment (ab90285, Native Human IgG FC fragment protein, GR3276085, Abcam, Cambridge, UK)) was administered by intraperitoneal injection directly before the radiotracer was injected (30μg per mouse, i.v, 10–13 MBq) in LNCaP-bearing BALB/cnu/nu mice (*n* = 3–4). Each animal was anesthetized at 0 and 48 h and 5 days post-injection, and SPECT imaging was conducted. Activity uptake in the heart was quantified as a percentage of the injected activity minus activity remaining in the tail.

### 4.4. Uptake Study

22Rv1 prostate cancer cells (4 × 10^6^ cells/mouse) were implanted in the right hind leg of 6–8-week-old male Balb/c nu/nu immunodeficient mice (Janvier, France). Tumors were allowed to grow for 3–4 weeks. On the day of the experiment, the average mice weight was 25.7 ± 1.5 g. Four mice were intravenously injected with 100 μL of ^177^Lu-hu5A10 (20 μg in 2%BSA-PBS, 130 kBq, 0.13 mM EDTA) in the tail vein. Seven days p.i., mice were euthanized using an overdose of anesthesia (20 μL of Ketalar Rompun per gram body weight: Ketalar (50 mg/mL; Pfizer, New-York, NY, USA), 10 mg/mL; Rompun, (20 mg/mL; Bayer, Leverkusen, Germany), followed by heart puncture and exsanguination with a syringe. Liver and tumor were collected and weighed, and their radioactivity concentration in the respective tissue was measured in a NaI(Tl) automated well counter (PerkinElmer, Waltham, MA, USA).

### 4.5. Autoradiography, Immunohistochemistry, and Immunofluorescence

Intra-tumoral distribution of radioactivity was studied using digital autoradiography. Specifically, cryosections (10 µm thickness) adjacent to sections used for immunohistochemical labeling were imaged using a Biomolex 700 Imager (Biomolex AS, Oslo, Norway) [33]. Corrections were applied for dead or miscalibrated detector strips and images reconstructed after a minimum of 1440 min of measurement. After autoradiography, sections were stained with hematoxylin and eosin and imaged using an automated whole slide imager (Carl Zeiss AG, Oberkochen, Germany).

Cryosections (10 µm) for PSA or Ki67 antibody single labeling was used to visualize their binding sites by means of HRP, DAB/H2O2-based reactions of adjacent sections. Whole labeled sections were slide scanned (Hammatsu). For simultaneous visualization of PSA and Ki67, tumor sections were incubated with a cocktail of primary and secondary species specific antibodies conjugated with fluorophores with separate emission spectra. A nuclear counterstain (4′,6-diamidino-2-phenylindole, DAPI) was used to visualize the cell nuclei, and confocal laser scanning microscopy (LSM 800, Zeiss, Germany) was used for the visualization of antibody binding sites. Details for PSA and Ki67 single immunohistochemistry and double immunofluorescence labeling are given in the Appendix A.

### 4.6. Therapeutic Efficacy

To evaluate the effect of chelate and chelate-to-antibody ratio, LNCaP tumor-bearing mice (*n* = 3–4 per group) were administered a single injection of 50 µg radiolabeled immunoconjugate of activities of 18 MBq with ^111^In-DOTA-hu5A10 chelate-to-antibody ratios of 3:1 and 6:1, or ^111^In-DTPA-hu5A10 with chelate-to-antibody ratios of 6:1 and 12:1. Syringe activity was measured before and after injection (Atom Lab 500 Dose Calibrator, Biodex Medical Systems Inc., Shirley, NY) to assure true injected activity. Body weight and tumor volume (external caliper measurement, V = 0.5 × length × width × width) were monitored 2–3 times per week for three weeks. Relative tumor size (RTS) was calculated as the natural logarithm of the fractional increase in volume relative to the tumor size at treatment start (logarithm of the relative tumor size (log(RTS)).

## 5. Conclusions

The present study demonstrated that the labeling method of choice had a substantial influence on binding affinity to the antigen, FcRn interaction, blood retention, tumor penetration, and internalization. Together, these parameters significantly affected the therapy outcome of an Auger-emitting radionuclide, emphasizing the importance of internalization and homogenous activity/absorbed dose distribution in tumors. Our results were especially interesting with regard to the interaction of the immunoconjugate with the FcRn, since this is a common route for internalization of antibodies, and also seems to influence the relationship between liver and tumor accumulation. Optimizing this feature further could improve the future use of hu5A10 in radiotheranostic applications.

## 6. Patents

Sven-Erik Strand, and David Ulmert are shareholders of DiaProst (Lund, Sweden), which holds patents for 5A10 targeting. Thuy A. Tran holds stock options in DiaProst.

## Figures and Tables

**Table 1:** The impact of different conjugation chelator-to-antibody ratios for two common bifunctional chelators (CHX-A″-DTPA or DOTA) on the binding kinetics to the target fPSA and FcRn, measured by SPR using a Biacore instrument. The results showed that labeling under increasing amounts of DOTA chelator affected the FcRn binding the most, whereas the same was true for fPSA binding with the DTPA chelator.

Antibody /Immunoconjugate	K_OFF_ (10^−6^ S^−1^)	Global Fit-Dissociation Curve (R^2^)	K_ON_ (10^6^ M^−1^ S^−1^)	K_D_ (10^−12^ M)	Global Fit-Association Curve (R^2^)	Fcrn Bound Per Antibody
hu5A10	1.28±0.05	1.00	1.7±0.06	0.75	0.98	1.8
hu5A10-DOTA						
3:1	1.56±0.03	0.99	0.68±0.03	2.31	0.98	0.8
6:1	1.13±0.04	0.99	0.53±0.02	2.12	0.98	0.5
12:1	1.24 ± 0.06	0.99	0.42 ± 0.02	2.92	0.98	0.1
hu5A10-DTPA						
3:1	2.47 ± 0.07	0.99	0.16 ± 0.01	15.09	0.99	1.9
6:1	2.52 ± 0.08	0.99	0.18 ± 0.01	13.68	0.98	1.9
12:1	2.26 ± 0.10	0.99	0.16 ± 0.01	14.49	0.98	1.7

**Table 2:** Ratio of chelator-to-antibody and radiochemical yield for hu5A10 conjugated with the chelators CHX-A″-DTPA or DOTA.

	Ratio of Chelator-to-Antibody	Radiochemical Yield (%)
***DOTA-hu5A10***		
**3:1**	0.8 ± 0.1	74 ± 3.0 (*n* = 2)
**6:1**	1.5 ± 0.7	70 ± 13.0 (*n* = 2)
**12:1**	2.4 ± 0.5	77 ± 6.0 (*n* = 2)
***CHX-A*** ***″-DTPA-hu5A10***		
**3:1**	1.4 ± 0.3	85 ± 4.0 (*n* = 3)
**12:1**	9.4 ± 2.1	89 ± 1.3 (*n* = 3)

**Table 3:** The tumor-to-organ ratios for ^111^In-CHX-A″-DTPA-hu5A10 and ^111^In-DOTA-hu5A10 with 3:1, 6:1, and 12:1 chelator-to-antibody molar ratios in LNCaP xenografted BALBC-nu mice.

**Tumour-to-organ of ^111^In-DOTA-hu5A10 for different chelator-to-antibody ratios**						
	**Blood**			**Liver**		
**Ratio**	**3**	**6**	**12**	**3**	**6**	**12**
24 h	0.40 ± 0.06	0.49 ± 0.13	0.54 ± 0.09	0.61 ± 0.18	0.65 ± 0.08	‡ 0.82 ± 0.08
48 h	0.55 ± 0.10	0.68 ± 0.10	0.56 ± 0.06	0.72 ± 0.13	† 0.96 ± 0.04	0.82 ± 0.15
120 h	0.57 ± 0.07	† 0.93 ± 0.16	0.81 ± 0.18	* 0.78 ± 0.06	† 1.27 ± 0.30	1.05 ± 0.19
**Tumour-to-organ of ^111^In-CHX-A″-DTPA-hu5A10 for different chelator-to-antibody ratios**						
48 h	1.1 ± 0.3	N/A	0.8 ± 0.5	0.8 ± 0.3	N/A	1.0 ± 0.3
168 h	3.0 ± 2.6	N/A	1.4 ± 0.3	0.3 ± 0.1	N/A	§1.7 ± 0.3

* Significant difference (*p* < 0.05) between 111In-DOTA-hu5A10 3:1 and 111In-DOTA-hu5A10 12:1. † Significant difference (*p* < 0.05) between 111In-DOTA-hu5A10 3:1 and 111In-DOTA-hu5A10 6:1. ‡ Significant difference (*p* < 0.05) between 111In-DOTA-hu5A10 6:1 and 111In-DOTA-hu5A10 12:1. § Significant difference (*p* < 0.05) between 111In-DTPA-hu5A10 3:1 and 111In-DTPA-hu5A10 12:1.

## Data Availability

The data will be at request at https://portal.research.lu.se/portal/sv/organisations-units/systemic-radiation-therapy(922e7906-fc50-4731-ad1c-579419b2212e).html (accessed on 30 April 2021).

## Appendix A

### Appendix A.1. Method

#### Appendix A.1.1. Conjugation

Humanized 5A10 (hu5A10) (Innovagen AB, Lund; 0.72 mg/mL in PBS pH 7.4, Lot No: 90475.24) was conjugated with either CHX-A″-DTPA (Macrocyclics) or p-SCN-Bn-DOTA (DOTA) (Macrocyclics) at a 3:1, 6:1, and 12:1 chelator-to-antibody molar ratio. Conjugation was done at room temperature in 0.07 M sodium borate buffer pH 9.2 (Sigma Aldrich, St Louis, MO, USA) and terminated after 8 h by size-exclusion chromatography on a NAP-5 column, equilibrated with 20 mL 0.2 M pH 5.5 ammonium acetate buffer (Sigma Aldrich). The immunoconjugates were eluted with 1 mL of the same ammonium aetate buffer. Aliquots were kept, if stored, at −20 °C before labeling.

#### Appendix A.1.2. Labeling Chemistry

The hu5A10 conjugated to CHX-A″-DTPA or p-SCN-Bn-DOTA, in a 3:1, 6:1 and 12:1 chelator-to-hu5A10 ratios were labeled with ^111^In (indium-111). ^111^InCl_3_ (20–30 MBq, Mallinkrodt Medical) was added to the respective conjugate diluted in 0.2M ammonium acetate buffer, pH 5.5, and the mixture was vortexed carefully. Reaction vials were incubated at 38 °C for 1 h (CHX-A″-DTPA-hu5A10) or 2 h (DOTA-hu5A10). The labeling efficiency of all radiolabeled conjugates was monitored using instant thin-layer chromatography strips, eluted with 0.2 M citric acid (Sigma Aldrich) and quantified with a Phosphor Imager system with Optiquant software (Perkin Elmer, Wellesley, MA, USA). All radiolabeled conjugates were purified from non-bound ^111^InCl3 using a size-exclusion NAP-5 column (Thermo Fischer Scientific, Waltham, MA, USA) equilibrated with PBS (Hyclone). For labeling with the β-emitter ^177^Lu (Curium, Stockholm), 30–50 μg of the conjugate was mixed with ^177^LuCl_3_ (15–25 MBq) and incubated at 38 °C with continuous vortexing for 30 min. Thereafter, the radiolabeled conjugate was purified using AMICON Ultra-0.5-centrifugal filter devices with a MWCO 30,000 Da (Millipore, Burlington, MA, USA). Radiochemical yield and purity of the radioconjugate was determined using silica-impregnated ITLC strips (150–771 DARK GREEN Tec-Control Chromatography strips, Biodex Medical Systems) eluted with 0.2 M citric acid and measured using the Cyclone Storage Phosphor System (PerkinElmer, Waltham, MA, USA).

#### Appendix A.1.3. In Vivo SPECT/CT Imaging

The SPECT imaging was performed using a preclinical SPECT/CT scanner (NanoSPECT/CT Plus, Mediso; Budapest, Hungary) equipped with the NSP-106 multipinhole mouse collimator used with energy windows of 20% centered over 172, and 245-keV energy peaks of ^111^In. Each imaging session was started by performing a short CT scan to acquire anatomical information. This was then followed by a 30–50 min SPECT scan for each animal. The scan data was reconstructed to a 3D image using HiSPECT software (Scivis GmbH, Göttingen, Germany) using standard settings. Images were analyzed using VivoQuant software (inviCRO Imaging Services and Software, Boston, USA), drawing regions by hand around the heart, the liver, and the tumor. Activity in blood (heart), liver, and tumor was minus anything remaining in the tail due to non-perfect injections.

The animals were anesthetized with isoflurane and restrained in a plastic tube with continuous administration of isoflurane. Vital signs were continuously monitored during imaging.

#### Appendix A.1.4. PSA and Ki67 Immunohistochemistry

Following SPECT imaging of ^111^In-DOTA-hu5A10 ratio 3:1, 6:1, and 12:1 groups, tumor tissue was collected from mice and frozen in cryomount (Histolab Products AB, Stockholm, Sweden) on dry ice, and the tissue was cryosectioned at 10 µm thickness.

Prior to immunohistochemistry, the cryosections were dried for 15 min at 37 °C. Sections were post-fixed in 4% paraformaldehyde (PFA). Tissues were then quenched for 10 min in 0.3% H2O2 (in PBS) at room temperature (RT), rinsed in PBS (3 × 3 min). Blocking was performed via incubation of sections in PBS containing 1% BSA and 0.05% Triton X-100 (PBSBSATX) for 30 min at RT.

Sections were then incubated with rabbit monoclonal primary antibodies made against PSA (ab240982, Abcam diluted 1:100–1:400) or against Ki67 (Clone SP6, Thermo Fischer Scientific) diluted for 90 or 120 min, respectively, at RT. Primary antibody specificity was tested by omitting the primary antibody incubation from the protocol in parallel sections to those incubated with primary antibodies.

Sections were rinsed in PBS (3 × 3 min) and incubated with HRP-conjugated secondary antibodies made in goat (Dako Cytomation, DAKO, DK, Agilent Technologies, or Jackson ImmunoResearch Laboratories, Inc., West Grove, PA), for 30 min at RT. All antibody incubations were performed in a moisture chamber. Following rinses in PBS (3 × 3 min), the immunoreaction was performed in a solution containing di-aminobenzidine (DAB, 0.5% in PBS) and H2O2 (0.1%) for 10 min at RT. After rinses in PBS (3 × 3 min) and distilled water, sections were counterstained with Mayers hematoxylin and then rinsed in distilled water. Sections were dehydrated in a graded alcohol serial (70–99.9%, 2 × 2) ended with Xylen (100% 2 × 5 min), and were then mounted and cover-slipped in Pertex (Histolab, Gtbg, Sweden).

The labeled whole sections were slide scanned using an automated whole slide imager (Hamamatsu NanZoomer S60, Hamamatsu, Japan).

#### Appendix A.1.5. Double Immunofluorescence Labeling

For the immunofluorescence, double staining of PSA and Ki67 sections (10 µm) were post-fixed in 4% PFA. Following incubation with blocking in PBSBSATX and rinses in PBS (3 × 3 min), sections were incubated with a mixture of primary antibodies, the monoclonal PSA made in rabbit used for immunohistochemistry and a Ki67 antibody made in rat (Invitrogen #14-5698-80). Antibody specificity was tested by omitting the primary antibody incubation from the protocol in parallel sections to those incubated with primary antibodies. Sections were then rinsed in PBS (3 × 3 min), incubated with a mixture of fluorophore-conjugated secondary antibodies (Jackson ImmunoResearch, West Grove, PA, USA) made in donkey against rabbit and rat IgGs (conjugated with AF488 or Rodamine RX, diluted 1:200 in PBSBSATX), for 30 min at RT. Antibody incubations were performed in a moisture chamber. Sections were then incubated in 4′,6-diamidino-2-phenylindole (DAPI) and rinsed in PBS, followed by mounting in anti-fade solution (ab104135, Abcam, Cambridge, UK).

Confocal laser scanning analyses of the fluorescence labeling were performed using a Zeiss LSM 800 microscope (Zeiss, Germany) with a x20 magnification lens.

### Appendix A.2. Results

#### Tumor and Normal Organ Uptake

Figure A1 shows the blood retention for ^111^In-DOTA-hu5A10 with 3:1 and 12:1 chelator-to-antibody molar ratios obtained from SPECT imaging performed at 0 h, 48 h, and 5 days post-injection. The results for FcRn blocking are also included. As can be seen, there was an increased blood concentration for ratio 12:1 compared to ratio 3:1. FcRn blocking enhanced the blood retention for both ratios, and there was a significant higher blood retention for ratio 3:1 after FcRn blocking. The blood, liver, and tumor uptake for ^111^In-CHX-A″-DTPA-hu5A10 with 3:1 and 12:1 chelator-to-antibody molar ratios and ^111^In-DOTA-hu5A10 with 3:1, 6:1, and 12:1 chelator-to-antibody molar ratios in LNCaP xenografted BALBC-nu mice can be seen in Table A1.

**Table A1:** The biodistribution for ^111^In-CHX-A″-DTPA-hu5A10 and ^111^In-DOTA-hu5A10 with 3:1, 6:1, and 12:1 chelator-to-antibody molar ratios in LNCaP xenografted BALBC-nu mice.

**^111^** **IN-DOTA-HU5A10**									
	Blood			Liver			Tumor		
**RATIO**	3	6	12	3	6	12	3	6	12
**0**	17.5 ± 2.0	20.4 ± 3.6	25.0 ± 4.0	15.5 ± 4.5	19.5 ± 3.91	11.4 ± 1.0	N/A	N/A	N/A
**24 H**	10.6 ± 0.5	9.7 ± 1.2	9.0 ± 1.5	7.3 ± 1.3	7.0 ± 0.6	5.9 ± 1.1	4.3 ± 0.8	4.6 ± 0.7	4.8 ± 0.7
**48 H**	9.4 ± 1.5	8.8 ± 1.5	11.1 ± 1.5	7.5 ± 1.5	6.1 ± 0.8	7.8 ± 1.7	5.1 ± 1.0	5.6 ± 0.9	6.2 ± 0.4
**120 H**	7.2 ± 1.0	7.5 ± 0.4	8.1 ± 1.2	5.9 ± 0.4	5.6 ± 0.4	6.1 ± 0.6	4.6 ± 0.7	6.6 ± 1.5	6.3 ± 0.4
**^111^** **IN-DTPA-HU5A10**									
	Blood			Liver			Tumor		
**RATIO**	3		12	3		12	3		12
**48 H**	9.5 ± 1.5		9.0 ± 2.0	13.9 ± 3.2		7.5 ± 0.9	10.4 ± 1.2		7.3 ± 1.9
**168 H**	2.5 ± 1.5		7.0 ± 1.1	17.8 ± 2.8		5.6 ± 0.9	5.4 ± 2.1		9.3 ± 0.9
